# Possible contribution of phosphate to the pathogenesis of chronic kidney disease in dolphins

**DOI:** 10.1038/s41598-023-32399-6

**Published:** 2023-03-29

**Authors:** Nourin Jahan, Hiroyuki Ohsaki, Kiyoko Kaneko, Asadur Rahman, Takeshi Nishiyama, Makoto Koizumi, Shuichiro Yamanaka, Kento Kitada, Yuki Sugiura, Kenji Matsui, Takashi Yokoo, Takayuki Hamano, Makoto Kuro-o, Takuya Itou, Miwa Suzuki, Keiichi Ueda, Akira Nishiyama

**Affiliations:** 1 Department of Pharmacology, Faculty of Medicine, Kagawa University, 1750-1 Ikenobe, Miki-cho, Kita-gun, Kagawa 761-0793 Japan; 2 Department of Medical Biophysics, Kobe University Graduate School of Health Science, Kobe, Japan; 3 Faculty of Pharmaceutical Sciences, Teikyo Heisei University, Tokyo, Japan; 4 Prime Hospital Tamashima, Kurashiki, Japan; 5 Laboratory Animal Facility, Research Center for Medical Sciences, Jikei University School of Medicine, Tokyo, Japan; 6 Division of Nephrology and Hypertension, Department of Internal Medicine, The Jikei University School of Medicine, Tokyo, Japan; 7 Multiomics Platform, Center for Cancer Immunotherapy and Immunobiology, Kyoto University Graduate School of Medicine, Kyoto, Japan; 8 Department of Nephrology, Nagoya City University Graduate School of Medical Sciences, Nagoya, Japan; 9 Department of Nephrology, Osaka University Graduate School of Medicine, Suita, Japan; 10 Division of Anti-Aging Medicine, Center for Molecular Medicine, Jichi Medical University, Tochigi, Japan; 11 Nihon University Veterinary Research Center, Nihon University, Fujisawa, Japan; 12 Department of Marine Science and Resources, College of Bioresource Sciences, Nihon University, Fujisawa, Japan; 13 Okinawa Churashima Foundation, Kunigami-gun, Japan

**Keywords:** Nephrology, Kidney

## Abstract

This study aimed to investigate whether phosphate contributes to the pathogenesis of chronic kidney disease (CKD) in dolphins. Renal necropsy tissue of an aged captive dolphin was analyzed and in vitro experiments using cultured immortalized dolphin proximal tubular (DolKT-1) cells were performed. An older dolphin in captivity died of myocarditis, but its renal function was within the normal range until shortly before death. In renal necropsy tissue, obvious glomerular and tubulointerstitial changes were not observed except for renal infarction resulting from myocarditis. However, a computed tomography scan showed medullary calcification in reniculi. Micro area X-ray diffractometry and infrared absorption spectrometry showed that the calcified areas were primarily composed of hydroxyapatite. In vitro experiments showed that treatment with both phosphate and calciprotein particles (CPPs) resulted in cell viability loss and lactate dehydrogenase release in DolKT-1 cells. However, treatment with magnesium markedly attenuated this cellular injury induced by phosphate, but not by CPPs. Magnesium dose-dependently decreased CPP formation. These data support the hypothesis that continuous exposure to high phosphate contributes to the progression of CKD in captive-aged dolphins. Our data also suggest that phosphate-induced renal injury is mediated by CPP formation in dolphins, and it is attenuated by magnesium administration.

## Introduction

As humanity enters a super-aging society, the number of patients with chronic kidney disease (CKD) is dramatically increasing^1^. Although the pathophysiology of age-related CKD is likely due to multiple factors^2^, the potential role of phosphate, which accelerates aging, has been recently attracting attention^3–5^. In mammals, a small increase in blood phosphate and calcium concentrations due to a transient increase after a meal may induce a risk of calcium phosphate precipitation^6,27^. However, when the blood phosphate concentrations increase, fibroblast growth factor 23 is secreted from bone and acts on the αKlotho and fibroblast growth factor receptor complex in the proximal tubule to promote phosphate excretion into the urine^7–9^. Furthermore, calcium phosphate precipitated in the blood is quickly adsorbed by fetuin-A and does not form large clumps^10^. The adsorbed calcium phosphate is dispersed in the blood as microscopic colloidal calciprotein particles (CPPs). CPPs sequester phosphate and calcium preventing ectopic calcification caused by the precipitation of calcium phosphate into extraosseous tissues^11^.

However, CPP agglomeration was recently shown to induce chronic inflammation and calcification in vascular tissues which contribute to the development of CKD^12,13^. Formed CPPs bind to toll-like receptor 4 expressed on tubular cells and are taken up into the tubules, causing tubular cell injury and inducing fibrosis of the renal interstitium^13^. Interestingly, in Dahl salt-sensitive hypertensive rats with normal blood phosphate concentrations, a phosphate binding agent does not alter serum phosphate concentrations but shows renoprotective effects by reducing urinary phosphorus excretion and suppressing CPP formation in renal tissue^12^. These data suggest that even if blood phosphate concentrations are maintained within the normal range, an increase in phosphate urinary excretion causes renal tissue injury by intratubular CPP formation^12,13^.

Excessive phosphate intake has been reported to induce CKD not only in humans but also in mammals across species^14,15^. Furthermore, the International Renal Interest Society, which was created to advance the scientific understanding of kidney disease in small animals, recommends feeding a low-phosphate diet^16^. However, no reports have examined the relationship between CKD and phosphate in cetaceans, which are marine mammals. In particular, the modern captive dolphin society is facing a super-aging population, and cases of deaths with declining kidney function have been often reported^17^. Indeed, the average lifespan of wild bottlenose dolphins is reported to be 20–30 years old^18^. However, some individuals have been reported to live to be older than 50 years old in captivity with proper treatment for infectious diseases and adequate nutrition^19^. Captive dolphins eat fish and squid as staple foods, both of which contain high levels of animal phosphate^20^. Therefore, we hypothesized that captive dolphins are at increased risk of developing CKD due to phosphate as they age. Clinical studies have indicated a possible relationship between the risk of phosphate-induced progression of CKD and serum magnesium concentrations^21^. We also hypothesized that magnesium attenuates phosphate-induced renal injury. To test these hypotheses, we investigated the presence of phosphate in components of renal necropsy tissue in an aged dolphin, and examined the toxicity of phosphate and the effects of magnesium in immortalized cultured dolphin tubular (DolKT-1) cells.

## Results

### Pathological findings and necropsy tissue sampling

An older dolphin (estimated to be > 50 years old) in captivity died of myocarditis and other causes. The heart, kidneys, lungs, liver, spleen, pancreas, testes, and diaphragmatic lymph nodes were removed. Dissected tissues were fixed with paraformaldehyde and part of the renal tissue were frozen for analyses. A histopathological examination by a pathologist showed that the mitral valve had numerous bacterial clusters and pyogenic inflammation, which indicated that bacterial endocarditis had occurred. Focal necrosis had occurred in the left ventricle and left kidney, which suggested infarction due to bacteria and thrombus from the mitral valve. The lungs showed focal interstitial fibrosis, which was thought to be an old change. Pyogenic enteritis was also observed in the intestines. The histopathological diagnosis was bacterial endocarditis, left ventricular infarction, left renal infarction, and suppurative enteritis, while there was no major change in the liver, spleen, adrenal gland, testes, or lymph nodes.

### Computed tomography (CT) scan

**Figure 1:**
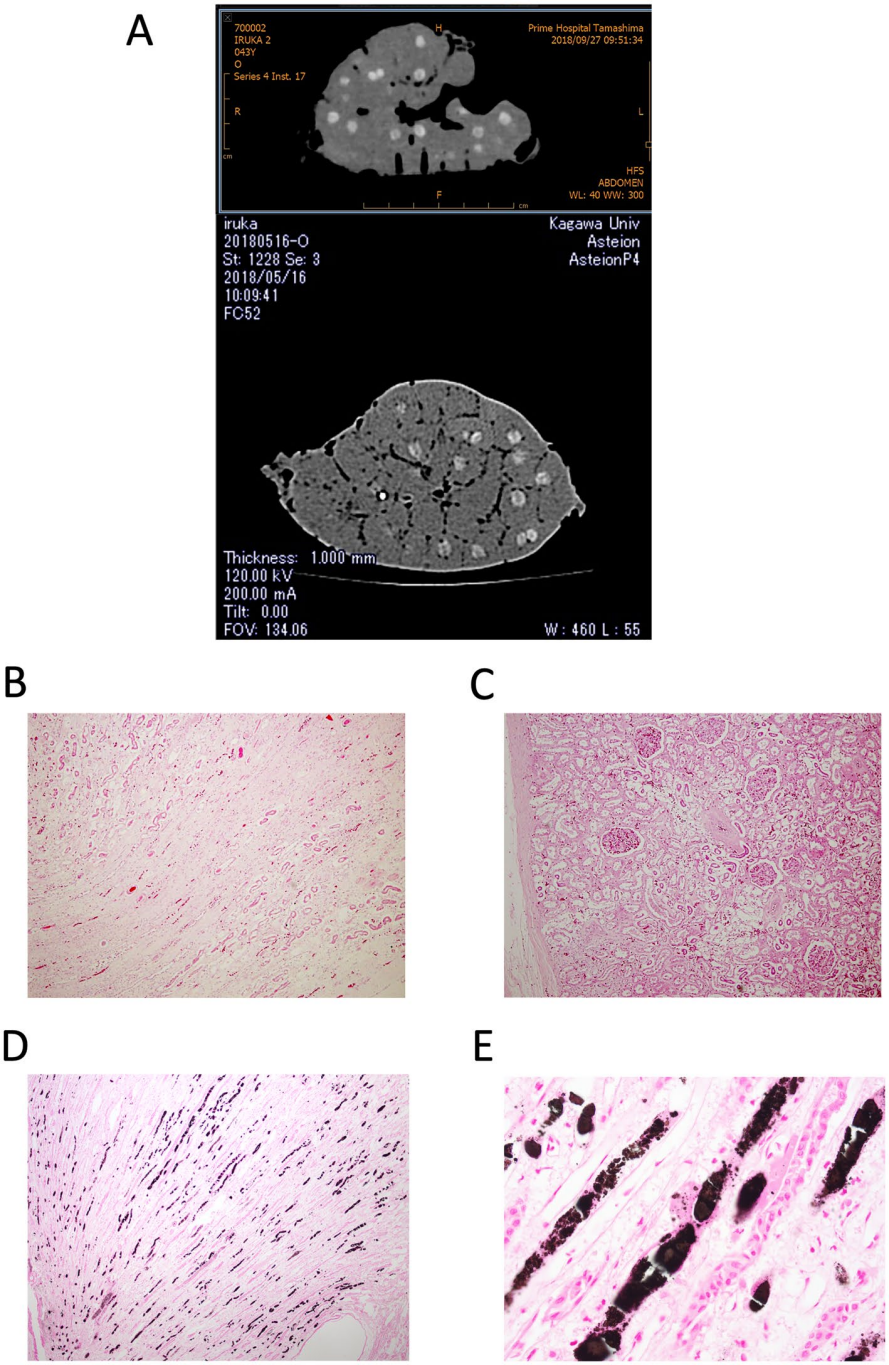
CT scan and histological findings in necropsy tissues in a dolphin. Representative CT image of the right kidney (**A**). A CT scan shows many high-density areas in the reniculi in the medullary portion of the kidney. Histology with HE staining (**B**,**C** original magnification, × 40 and 100, respectively) and Von Kossa (**D**,**E** original magnification, × 100 and 400, respectively) staining, respectively. There is no obvious abnormality in the renal tissues or vessels except infarcted area, but minor glomerular sclerosis and interstitial fibrosis can be seen. Von Kossa staining is positive in part of the medullary region. *CT* computed tomography, *HE* hematoxylin and eosin.

A computed tomographic (CT) scan of the removed right kidney showed many high-density areas in the reniculi in the medullary portion, which could be considered calcification (Fig. 1A). Similarly, high-density areas were observed in a series of cross-sectional movies of the reniculi taken using micro-CT (Supplemental Movie Data).

### Micro area X-ray diffractometry and infrared absorption (IR) spectroscopy

Dried medullary tissues were analyzed with microbeam X-rays and IR spectroscopy. As shown in Supplementary Figs. S1A and S1B, micro area X-ray analysis determined high-density area to contain hydroxyapatite (calcium phosphate). On the other hand, IR spectroscopy data showed that absorption of reniculi tissues of high-density area in the medulla was observed at 1457, 1040, 873, 606, and 567 cm^−1^ (green line, Supplementary Fig. S1C). When the absorption wave number was compared with reported IR data, this renal tissue was shown to contain hydroxyapatite. Interestingly, in tissues of normal- or low-density area, absorption at 1040 cm^−1^ was smaller, and those at 873, 606, and 567 cm^−1^ was not observed (red line). These data indicate that reniculi medullary tissues contain hydroxyapatite (calcium phosphate).

### Histological findings

Hematoxylin and eosin (HE) staining of formalin-fixed renal tissue showed no obvious abnormalities in the renal tissue or vessels except infarcted area, but some parts of the glomerulus and tubulointerstitium appeared to have sclerosis and fibrosis, respectively (Fig. 1B,C). However, Von Kossa staining showed positive staining in a part of the medullary region (Fig. 1D,E).

### Effects of phosphate and magnesium on DolKT-1 cell morphology

**Figure 2:**
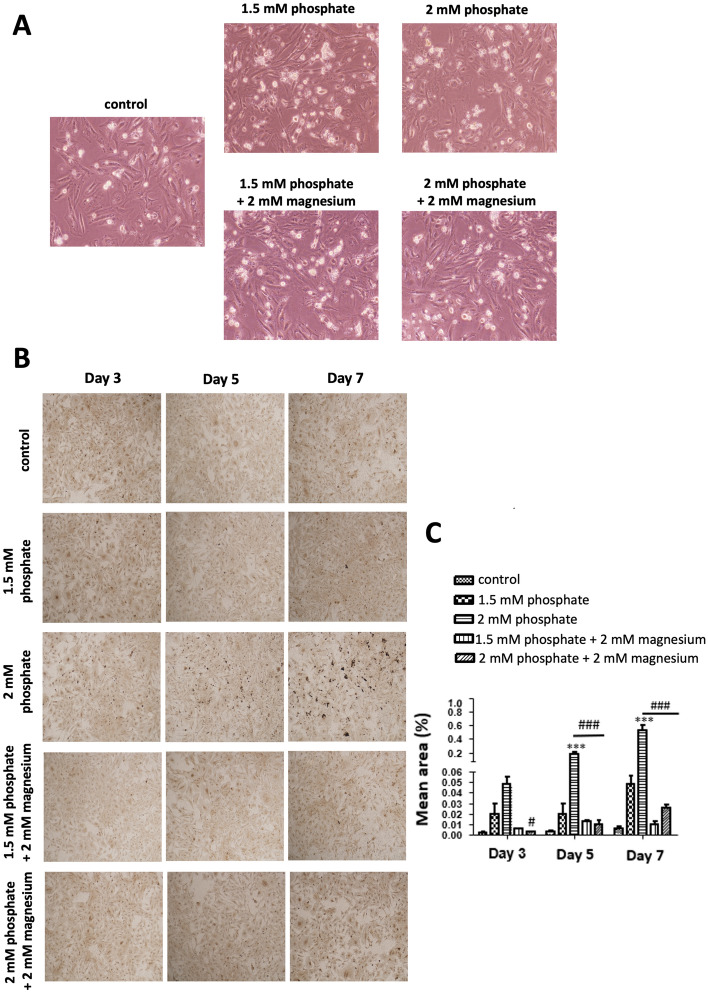
Effects of phosphate and magnesium on DolKT-1 cell calcification. Morphological features of cells exposed to 0.9 mM phosphate and 0.8 mM magnesium (control), 1.5 mM phosphate, 2 mM phosphate, 1.5 mM phosphate + 2 mM magnesium, and 2 mM phosphate + 2 mM magnesium (**A**). The images were taken after 72 h of exposure. Magnification, × 42. Von Kossa staining shows calcium deposition in each experimental group at 3, 5, and 7 days of exposure. Images are representative of three separate experiments (**B**). Quantitative analysis of Von Kossa staining was performed using morphometric analysis by Image J (Image J bundled with 64-bit Java 8; https://imagej.nih.gov/ij/download.html?fbclid=IwAR060tXklMhoVowfvHzwq9sNxID7_IkQBrPxqD6Ej4fN68jqlGbYma40eFc) on the pooled results of three separate experiments (**C**). n = 4 for each group in each culture condition. ***P < 0.001 vs. control; ^**#**^P < 0.05, ^###^P < 0.001 vs. 2 mM phosphate, respectively. *DolKT-1 cells* Dolphin proximal tubular cells.

DolKT-1 cells were exposed to two different concentrations of phosphate at 1.5 and 2 mM. The treatment with 1.5 mM phosphate resulted in moderate shrinkage and a rounded shape of cells. However, 2 mM phosphate for 72 h induced dramatic morphological changes, such as cell shrinkage, rounding, and loss of cell attachment to the substratum compared with the control treatment (0.9 mM phosphate and 0.8 mM magnesium). We also examined the effect of magnesium on phosphate-induced DolKT-1 cellular changes. We found that pretreatment with 2 mM magnesium completely abolished the phosphate-induced morphological changes (Fig. 2A).

### Effects of phosphate and magnesium on DolKT-1 cell calcification

Von Kossa staining showed that phosphate dose-dependently increased calcium deposition in DolKT-1 cells (Fig. 2B). Dense calcification was observed after 2 mM phosphate exposure for 3–7 days. Morphometric analysis of calcium deposition showed that phosphate-induced calcification was completely abolished by the addition of magnesium. Concomitant treatment with magnesium caused a significant reduction in calcification within 3 days of intervention (P < 0.05, Fig. 2C). Similar inhibition was also observed on day 5 and 7 (P < 0.001, respectively).

### Effects of phosphate and magnesium on DolKT-1 cell viability

**Figure 3:**
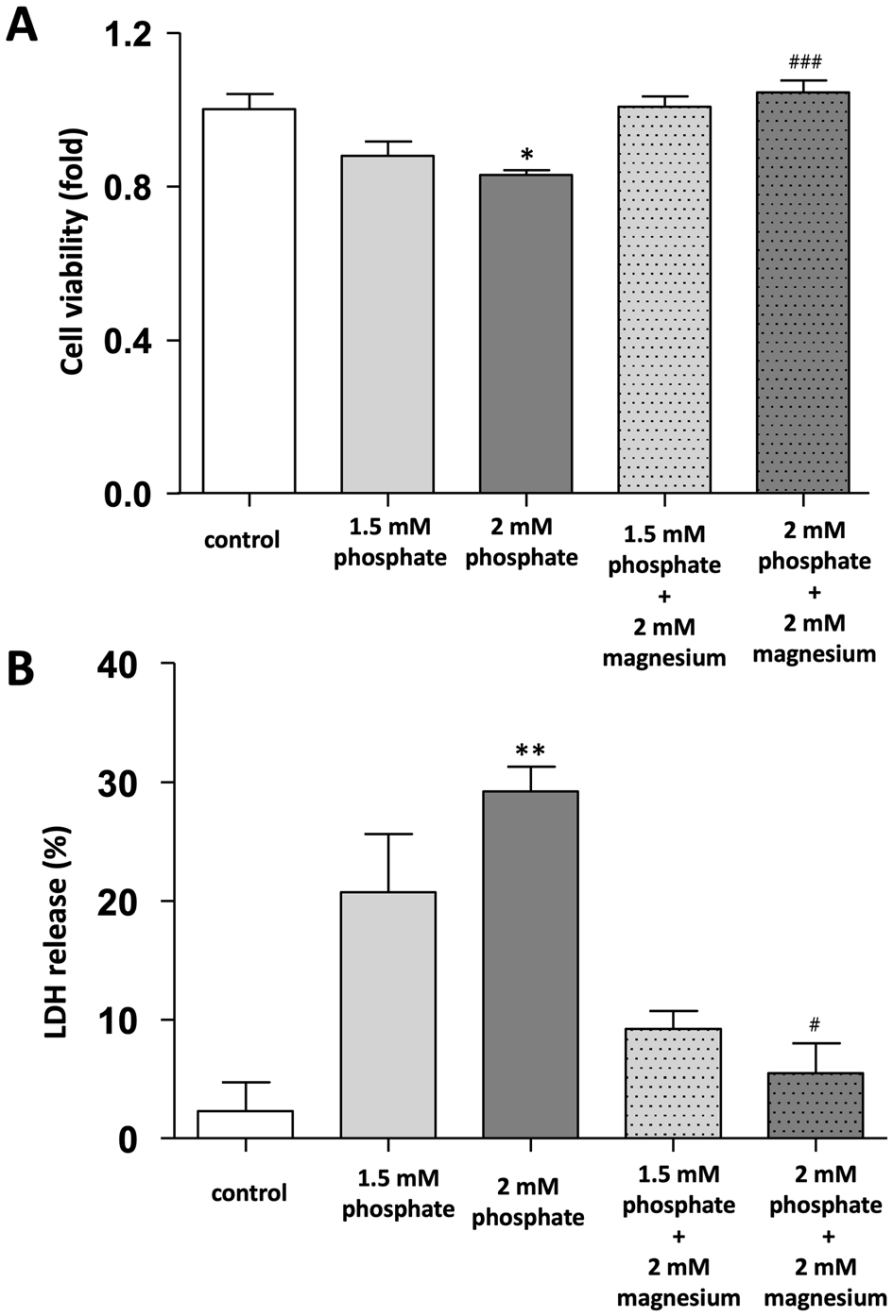
Effects of phosphate and magnesium on DolKT-1 cell viability and injury. Cell viability was quantified by the WST-1 assay after 48 h of exposure with various experimental groups and expressed as the fold change with the live cells in the control group (**A**). n = 4 or 5 for each group. Cytotoxicity was detected by LDH assay after 48 h of exposure (**B**). n = 5 for each group. *P < 0.05, **P < 0.01 vs. control; ^#^P < 0.05, ^###^P < 0.001 vs. 2 mM phosphate, respectively. *WST-1* water-soluble tetrazolium salt-1, *LDH* lactate dehydrogenase.

The cell viability was analyzed by the water-soluble tetrazolium salt-1 (WST-1) assay following 48 h of exposure to phosphate and concomitant treatment with magnesium. Phosphate dose-dependently decreased cell viability in DolKT-1 cells (P < 0.005, Fig. 3A). However, concomitant treatment with magnesium markedly attenuated the phosphate-induced reduction in cell viability.

### Effects of phosphate and magnesium on DolKT-1 cellular injury

Cellular injury was evaluated by lactate dehydrogenase (LDH) release in DolKT-1 cells (Fig. 3B). Treatment with phosphate dose-dependently elevated LDH release (P < 0.05). However, concomitant treatment with magnesium markedly attenuated the phosphate-induced LDH release. These data suggested that magnesium protected the DolKT-1 cells against phosphate-induced cytotoxicity.

### Effects of phosphate on DolKT-1 cell apoptosis

**Figure 4:**
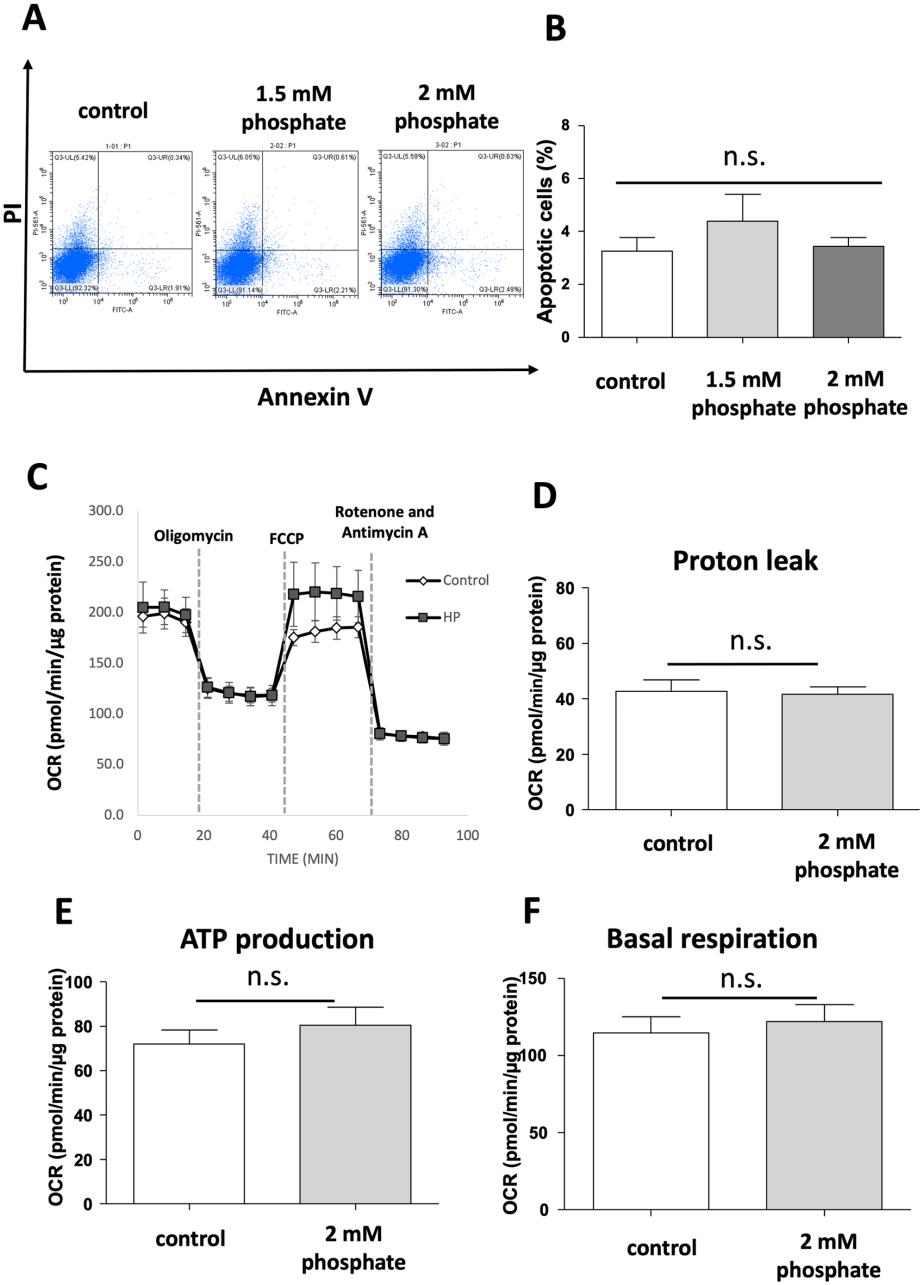
Effects of phosphate on DolKT-1 cell apoptosis and mitochondrial function. Apoptotic cells were determined using flowcytometry with annexin V/PI double staining (**A**). Data were analyzed by CytExpert ver. 2.3 (https://www.beckman.com/flow-cytometry/research-flow-cytometers/cytoflex/software). The bar graph shows the percentage of apoptotic cells in each experimental group, n = 4 for each group (**B**). DolKT-1 cells were pretreated with 2 mM phosphate for 36 h and mitochondrial function was measured by flux analysis. Oxygen consumption rate (**C**), proton leak (**D**), ATP production (**E**), and basal respiration (**F**). n = 2 for each group. *n.s* not significant, *PI* propidium iodide.

To detect apoptosis, flow cytometry was performed by counting annexin V- and propidium iodide (PI)-stained positive cells. The proportion of apoptotic cells (annexin V-positive and PI-negative cells indicated early apoptosis, and annexin V-positive and PI-positive cells indicated late apoptosis) was not significantly changed by phosphate or magnesium (Fig. 4A,B).

### Effects of phosphate on mitochondrial function in DolKT-1 cells

Flux analysis was performed to investigate mitochondrial dysfunction and damage in DolKT-1 cells. The oxygen consumption rate was measured (Fig. 4C). Treatment with 2 mM phosphate did not result in any significant difference in proton leak, which is an indicator of mitochondrial damage (Fig. 4D), ATP production (Fig. 4E), or basal respiration (Fig. 4F).

### Effects of CPPs on DolKT-1 cell damage

**Figure 5:**
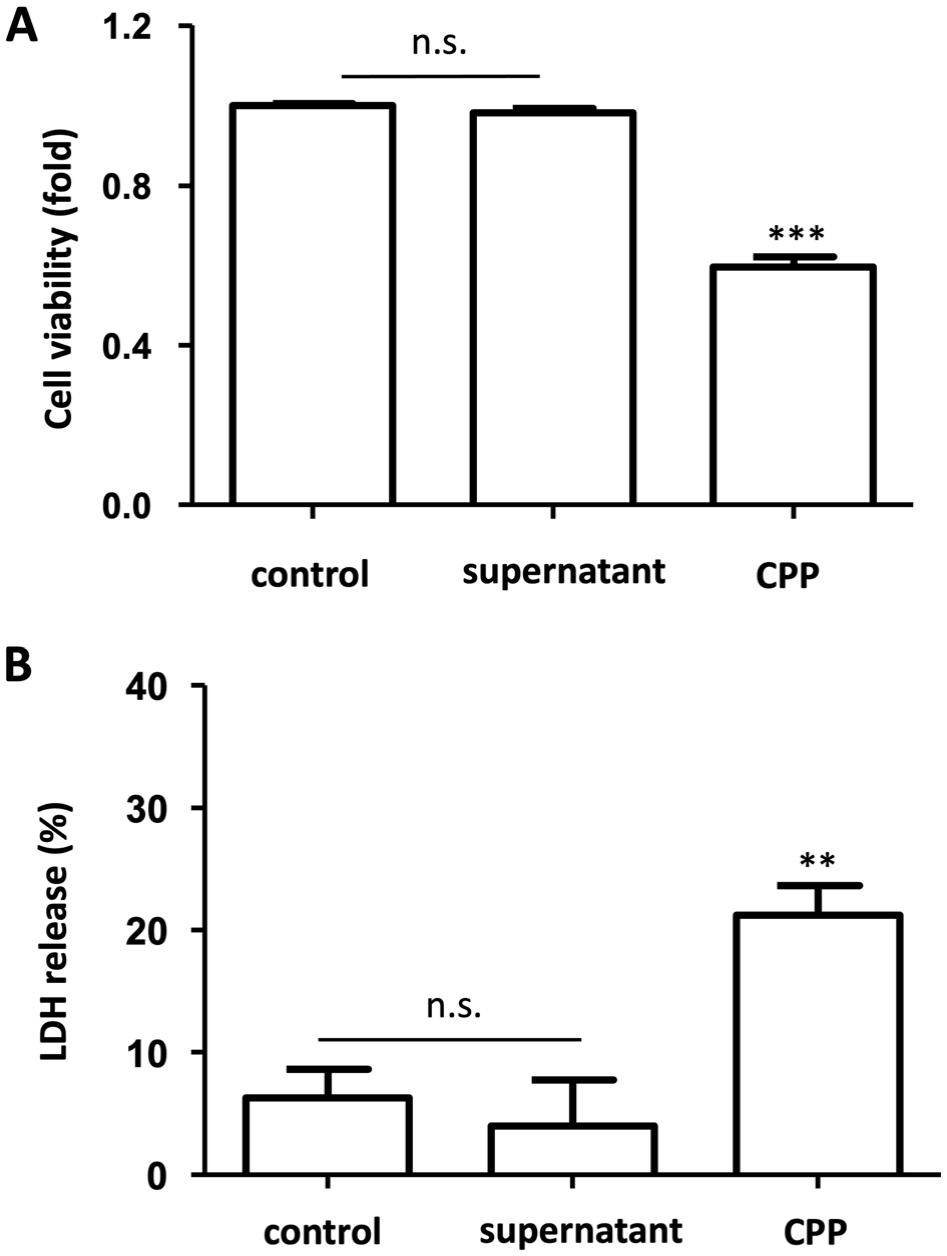
Effects of CPPs on DolKT-1 cell damage. Cell viability of DolKT-1 cells was measured by using a WST-1 method. Cells were treated for 24 h with the control medium, the supernatant of the high phosphate plus high calcium-containing medium after centrifugation, or CPP suspension (**A**). n = 6 for each group. LDH release was measured after treatment with control solution, supernatant, or CPPs for 24 h (**B**). n = 5 for each group. **P < 0.01, ***P < 0.001 vs. control. *CPP* calciprotein particles.

To investigate whether phosphate-induced cell injury is mediated through CPP formation, we centrifuged the high phosphate- and calcium-containing medium at 16,000×*g* for 2 h to precipitate the CPPs and removed the supernatant. We, then treated the cells with supernatant and CPPs for 24 h. We found that CPPs significantly decreased cell viability (P < 0.0001, Fig. 5A) and increased LDH release (P < 0.05, Fig. 5B), while these changes were not observed by treatment with only the supernatant. These data suggested that CPPs were responsible for causing cell damage.

### Effects of magnesium on CPP-induced DolKT-1 cell damage

**Figure 6:**
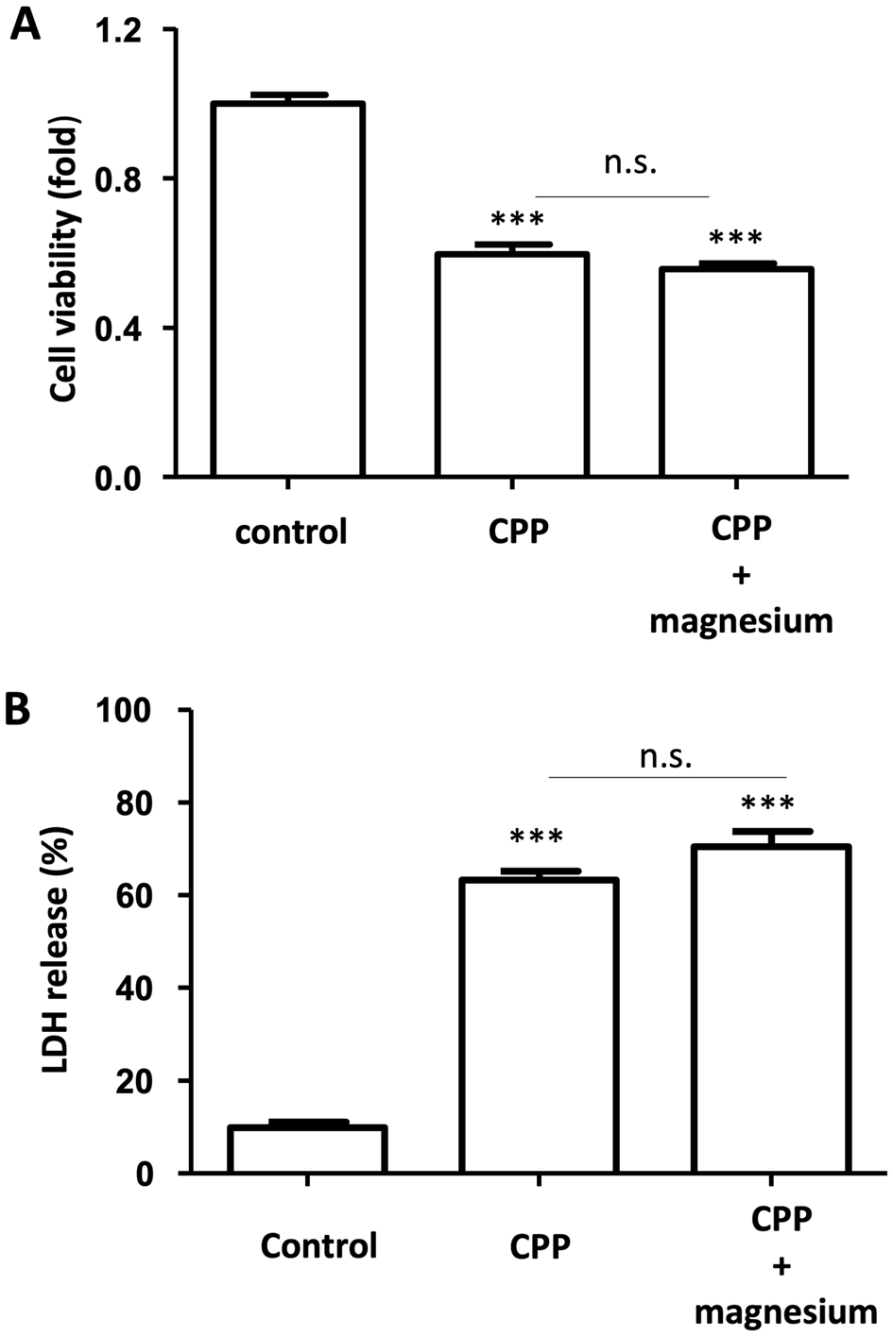
Effects of magnesium on CPP-induced DolKT-1 cell damage. The cell viability was measured by the WST-1 method after 48 h of exposure in the experimental groups (**A**). Data are expressed as the fold change level of the live cells in the control group. n = 8 for each group. LDH release was measured in the control, CPP, and CPP + magnesium groups after 24 h of exposure (**B**). n = 5 for each group. ***P < 0.001 vs. control.

The administration of CPPs resulted in a significant reduction in cell viability (P < 0.0001, Fig. 6A). Supplementation with magnesium did not attenuate the CPP-induced reduction in DolKT-1 cell viability. The administration of CPPs significantly increased LDH release (P < 0.0001, Fig. 6B), which was not changed by treatment with magnesium.

### Effects of magnesium on CPP formation

To investigate the mechanism by which magnesium attenuates phosphate-induced cell injury, we evaluated CPP formation by measuring the absorbance when concentrations of high phosphate (5 mM), high calcium (6 mM), and high magnesium (2 or 5 mM) were mixed together in the absence or presence of fetal bovine serum (FBS). In the presence of FBS, the 5 mM phosphate + 6 mM calcium group showed increased CPP formation from the initial time points and gradually increased over time. At each time point, magnesium significantly decreased CPP formation induced by high phosphate and calcium concentrations (Supplementary Fig. S2A). However, magnesium did not change CPP formation in the absence of FBS (Supplementary Fig. S2B).

## Discussion

During high phosphate loading, CPP formation in the tubular lumen is greatly affected by the amount of phosphate reached per single nephron, independent of changes in blood phosphate concentrations^22^. In humans, the number of nephrons declines with age, with approximately 50% fewer nephrons in the 70s than in the 20s^23^. There have been no reports examining changes in the nephron number in detail in dolphins. However, a diet of phosphate-rich fish and squid for many years may increase the risk of intratubular CPP formation, accompanied by a reduction in the number of nephrons as they age. In the present study, we analyzed renal tissue from an older dolphin that had died, and its renal function had been diagnosed as normal by monthly blood tests. Surprisingly, the medullary portion of reniculi tissue showed marked calcified lesions due to calcium phosphate (hydroxyapatite) accumulation. Further in vitro experiments using cultured dolphin tubular cells also showed that CPPs actually injured dolphin tubular cells. To the best of our knowledge, this is the first report to show that the risk of phosphate-induced CKD increases with aging in captive dolphins.

In the captive dolphin in which a necropsy was performed, a pathologist diagnosed the cause of death as bacterial endocarditis, and identified the site of infarction in the left ventricle and kidney caused by the resulting bacteria and blood clots. Blood tests showed that blood urea nitrogen, creatinine, and phosphate concentrations were within the normal range until just before death (Supplementary Table S1). However, a detailed analysis using CT scans showed the presence of a high-density area in many reniculi in medullary tissue, which strongly indicated calcification of renal tissue. Interestingly, micro-domain X-ray diffraction analyses of the medullary tissue showed that it contained a large amount of hydroxyapatite. Further analyses by IR spectroscopy confirmed that the peak observed in the calcified area was hydroxyapatite. Histological examination showed Von Kossa-positive staining with calcification in renal medulla.

In the present study using immortalized cultured dolphin tubular DolKT-1 cells, we found that phosphate changed cellular morphology and calcium deposition, which were associated with a marked reduction in cell viability. Moreover, phosphate also caused cytotoxicity as indicated by LDH release in the culture medium. To further determine the mechanism responsible for the phosphate-induced cytotoxicity, we examined the effects of phosphate on cell apoptosis and mitochondrial function. However, phosphate did not alter cell apoptosis or mitochondrial function in dolphin tubular cells, suggesting that alternative mechanisms may be involved. In this regard, Fujimura et al.^24^ reported that phosphate-induced mitochondrial damage in human proximal tubular cells was suppressed by the appropriate function of autophagy. Recent studies in human proximal tubular HK-2 cells have shown that phosphate is involved in cell damage by activating multiple intracellular signaling pathways^13,25,26^. Further detailed studies are required to determine if similar molecular mechanisms are involved in dolphin tubular cell injury. In mammals, a small increase in blood phosphate and calcium concentrations due to a transient increase after a meal may induce a risk of calcium phosphate precipitation^6,27^. To prevent amorphous calcium phosphate precipitates from being in a crystallized form, which may be responsible for ectopic calcification, a mineral-binding protein fetuin-A binds together and forms soluble colloidal particles called CPPs^10^. Although the covering of mineral crystals by fetuin-A is thought to prevent ectopic calcification, CPPs undergo a topological change from amorphous CPP1 to crystalline CPP2 in a high-phosphorus environment^28^. Several studies have shown that CPP2 formation induces calcification, inflammation and oxidative stress^29^, and renal injury^26^. Recently, Kunishige et al.^26^ showed that CPPs were incorporated into human proximal tubular HK-2 cells and caused the disruption of lysosomal homeostasis, autophagic flux, and plasma membrane integrity without causing oxidative stress. Furthermore, Shiizaki et al.^13^ reported that CPPs formed in the proximal tubule lumen induced inflammation and cell death, leading to tubular injury and interstitial fibrosis. In the present study, supernatant centrifuged from the culture medium did not alter cell viability or LDH release during treatment with high phosphate concentrations, while purified CPPs induced cell viability loss and cytotoxicity. These data indicate that dolphin tubular damage caused by high phosphate concentrations is mediated, at least in part, by formed CPPs.

Recent cohort studies in patient with CKD have shown that low serum magnesium concentrations increase the risk of end-stage kidney disease caused by high serum phosphate concentrations^21^. This finding suggests a close relationship between magnesium deficiency and phosphate renal toxicity. Studies using 5/6 nephrectomized mice showed that a low-magnesium diet reduced α-klotho expression in the kidney and significantly worsened tubulointerstitial fibrosis induced by a high-phosphate diet^30^. In the present study, phosphate-induced changes in cultured dolphin tubular cell morphology, Von Kossa-positive calcification, and cell damage were prominently suppressed by magnesium administration. These data suggest that magnesium has protective effects against phosphate-induced dolphin tubular cell injury. However, our data also showed that these effects of magnesium were not observed in CPP-induced dolphin tubular cell injury. Interestingly, the addition of magnesium to phosphate significantly inhibited the formation of CPPs, while magnesium administration to CPPs did not affect the concentration of CPPs per se. These data support the hypothesis that magnesium attenuates phosphate-induced injury of dolphin tubular cells through the inhibition of CPP formation.

Recently, an open-label, randomized, controlled trial by Sakaguchi et al.^31^ showed that magnesium oxide administration improved coronary artery calcification in patients with renal failure. Nevertheless, magnesium-induced intervention human clinical studies on the improvement of the prognosis of CKD have not been conducted yet. Aquatic geochemistry studies have indicated that magnesium stabilizes the amorphous calcium phosphate phase possibly resulting from the combination of multiple mechanisms. In particular, the direct precipitation of apatite in seawater upon the addition of dissolved inorganic phosphate is inhibited by magnesium ions^32^. In the body, hydroxyapatite- and protein-containing CPPs are major drivers of calcification^29,33^. The transition from calcium- and phosphate-containing amorphous or primary CPP1 towards crystalline or secondary CPP2 is key in the development of calcification^28,33,34^. Additionally, magnesium may delay the formation of secondary CPP2, thereby preventing phosphate-induced calcification^35,36^. The present study suggests that, as in humans, magnesium may be effective to attenuate the progression of renal injury in dolphins. Therefore, future studies need to carefully investigate the benefit of magnesium administration to captive dolphins with CKD complications.

A limitation of this study is that we could not examine the cytotoxic effects of CPP1 and CPP2 in DoIKT-1 cells and the effect of magnesium on CPP1 and CPP2 formation separately. The necessary technology to measure CPP1 and CPP2 separately by combining different characterization techniques, such as turbidimetry, dynamic light scattering, infrared spectroscopy, and scanning electron microscopy, is not available at our institution. Currently, synthesizing pure CPP1 and CPP2 is also difficult. In the future, we will establish such technologies to examine these effects.

In conclusion, we analyzed necropsy renal tissue from an older dolphin with normal renal function and found that the medullary portion of reniculi tissue had marked calcified lesions due to calcium phosphate (hydroxyapatite) accumulation. Further in vitro experiments in cultured dolphin tubular cells showed that phosphate-damaged tubular cells through the formation of CPP. The Inhibition of CPP formation by magnesium administration significantly attenuated the phosphate-induced dolphin tubular cell injury. These data suggest that dolphins are at increased risk of developing CKD due to phosphate accumulation in the kidney as they age. Further studies are required to determine the possible relationship between renal function and urinary phosphate excretion or plasma CPP concentrations in aged captive dolphins.

## Methods

In the present study, we did not perform any experiments on humans and use of human samples. This study was conducted using veterinarian-observed dolphin health examination data and postmortem necropsy samples. Therefore, no painful medication, anesthesia, or sampling was performed on the live dolphin.

Experimental protocols (Protocol No. 21601) were approved by the Animal experimentation Ethics Committee at Kagawa University. All procedures in this study were carried out in compliance with the Fundamental Guidelines for Proper Conduct of Animal Experiments and Related Activities in Academic Research Institutions under the jurisdiction of the Ministry of Education, Culture, Sports, Science, and Technology as well as WAZA (World Association of Zoos and Aquariums). We also followed the ethical guidelines for the Conduct of Research on Animals by Zoos and Aquariums and the guidelines for animal experiments of Kagawa University.

All dolphins in Ocean Expo Park (OEP; Kunigami-gun, Japan) have been housed following category 1 animal handling business, which is standard for housing and exhibiting animals approved by Okinawa Prefecture (OEP; No. 643), as previously described^37^ in detail. The health of the dolphins was monitored monthly by veterinarians on the basis of blood chemistry and behavior. Dolphins were maintained in outdoor pools with sea water sterilized by pressure filtration using polyester fiber and silica sand.

### Sample collection for necropsy and CT scan

A male Indo-Pacific bottlenose dolphin, estimated to be approximately 50 years old, has been kept at OEP for 43 years since May 1, 1975. Renal function remained in the normal range until 2015 (Supplementary Table S1). Beginning in October 2017, this dolphin presented with symptoms, such as anorexia, and blood tests suspected infection. Therefore, antibiotics were administered. During the following 6 months, the dolphin alternated between anorexia and temporary recovery. However, the general condition worsened later, and despite treatment with antibiotics, corticosteroids, and intravenous drip, the dolphin developed respiratory failure and died on March 22, 2018. A complete necropsy was immediately performed, and several tissues including the kidneys were dissected. Dissected tissues were fixed with paraformaldehyde and part of the right kidney tissues were frozen for analyses.

Thereafter, CT (Asteion Super 4 Edition, Toshiba Medical Systems Co., Ohtawara, Japan and SOMATOM Scope, Siemens Healthineers Japan, Tokyo, Japan) and micro-CT scans (Latheta LCT-200, Hitachi Aloka Medical Ltd., Tokyo, Japan) were performed on the removed right kidney and reniculi, respectively, and some of the tissues were dried completely in a dryer for 3 days. Dried samples were used for analysis by microarea X-ray diffractometry and IR spectrometry.

### Histological analysis

Renal tissues were dissected and fixed with 10% buffered paraformaldehyde, embedded in paraffin, and sectioned into 3-μm-thick slices. The sections were then stained with hematoxylin and eosin (HE), or Von Kossa reagent^38^.

### Micro area X-ray diffractometry

Dried medullary tissues were analyzed with microbeam X-rays at several locations. In this study, micro-area X-ray diffractometer (RINT-RAPID II Rigaku, Tokyo, Japan) with a microscope was used as previously described^39^. The analytical conditions were as follows: target, Cu; filter, Ni; voltage, 40 kV; current, 36 mA; and collimator diameter, 100 µm. The diffraction patterns obtained were compared with the data that were registered in the database of the Joint Committee on Powder Diffraction Standards (JCPDS).

### IR spectroscopy

After X-ray analysis, tissues were ground to powder and, then analyzed with IR spectroscopy^40^. IR spectra of the powders were recorded using a KBr tablet and IR spectrometer (FT/IR-4200 Jasco Tokyo, Japan).

### Cell line

DolKT-1 cells were obtained from the Department of Marine Science and Resources, College of Bioresource Science, Nihon University, Kanagawa, Japan. The establishment procedure of this cell line was described elsewhere^41^.

### Cell culture

DolKT1 cells were cultured in Dulbecco’s modified eagle medium (DMEM) (Cat# 11885084; Gibco, Grand Island, NY) supplemented with 10% FBS (Nichirei Biosciences, Tokyo, Japan), 1% insulin-transferrin-selenium (ITS-G 100X, # 41400045, Thermo Fisher, Waltham, MA), 50 U streptomycin/mL and 50 mg penicillin/mL (Life Technologies, Van Allen Way Carlsbad, CA). The cells were grown at 37 °C in a humidified atmosphere with 5% CO_2_. We tested for mycoplasma on a regular basis. The cells were passaged at 90% confluency and exposed to different experimental conditions. The culture medium was changed with 1% FBS (if not mentioned separately) for 18 h before the experiments. All experiments were performed between passages 24 and 30.

### Treatment protocol

DMEM (phosphate and magnesium concentrations in the medium were 0.9 and 0.8 mM, respectively) with 1% FBS was set as the control. In the treatment groups, the control medium was supplemented with NaH_2_PO_4_ and Na_2_HPO_4_ at a 1:2 proportion to reach the final phosphate concentration of 1.5 mM phosphate, and 2 mM phosphate respectively. MgCl_2_ was used to raise the magnesium concentration up to 2 mM. The pH of the medium was maintained at 7.4 in each case. The experiments conducted in this study were repeated at least three times.

### Cell morphological analysis

Cell morphology was examined by using an inverted light-phase contrast microscope (Olympus FSX100; Olympus Corporation, Center Valley, PA).

### Von Kossa staining

Calcium deposition of the cultured cells was detected by Von Kossa staining. The cells were seeded in the 24-well plates at 1.5 × 10^5^ cells/mL with a regular DMEM culture medium. After reaching 70% confluence, the cultured medium was switched to 5% FBS-containing medium for 18 h. Subsequently, cells were cultured for 3, 5, or 7 days in accordance with our treatments containing 5% FBS. With regard to staining, we mostly followed a previously reported protocol^42^ with some modifications. Briefly, the cells were washed two times with phosphate-buffered saline (PBS) followed by fixation with 4% paraformaldehyde for 20 min. Then again cells were washed with PBS two times and once with water. After this washing, 2% silver nitrate solution was added and exposed to ultraviolet light for 30 min. After washing again with water, 5% sodium thiosulfate was added and kept for 3 min. After washing with water, hematoxylin was added for 10 min to counterstain the nuclei. Finally, after three times washing with water, calcification was observed by an Olympus FSX100 microscope (Olympus Corporation) at a magnification of × 42.

### Cell viability

Cells were seeded on 24-well tissue culture plates at 1.5 × 10^5^ cells/mL and allowed to grow up to 70% confluence, and then switched to 1% FBS medium for 18 h. The intervention was performed for 48 h, and cell viability was then measured using a WST-1 assay kit in accordance with the manufacturer's protocol (Takara Bio, Otsu, Japan). Briefly, 50 µL of WST-1 reagents were added to each well of 500 µL of cell culture medium and incubated for 2 h, and the absorbance was measured with a microplate reader (Corona Multi Grating Microplate Reader SH-9000Lab; Hitachi High-Tech Science Corporation, Tokyo, Japan) at a wavelength of 480 nm.

### LDH assay

LDH concentrations in the medium were measured as an indicator of cell injury. An LDH cytotoxicity assay kit (Item no. 601170, Cayman Chemical, East Ellsworth Road Ann Arbor, MI) was used in accordance with the manufacturer's protocol. Briefly, the cell supernatant was removed after centrifugation, mixed with LDH reaction solution (reagents provided with the kit), and incubated at 37 °C for 30 min. The absorbance was measured at 490 nm with a microplate reader (Corona Multi Grating Microplate Reader SH-9000Lab, Hitachi High-Tech Science Corporation).

### Apoptosis assay

To detect apoptosis, flow cytometry was performed by counting annexin V- and PI- positive cells in line with the manufacturer’s instructions (catalog no #ab14085; Abcam, Cambridge, UK). Briefly, cells were cultured for 48 h in accordance with treatments and then trypsinized to obtain single cells. After washing with PBS twice, the cells were resuspended in a binding buffer with annexin V and PI for 5 min at room temperature. Apoptosis-positive cells were detected by using flow cytometry (BD FACS Canto II; BD Biosciences, San Jose, CA). Cells that were annexin V-positive and PI-negative were considered apoptotic. At least 20,000 cells were analyzed in each sample.

### Mitochondrial oxygen consumption

Mitochondrial oxygen consumption was measured by the Seahorse XFp Analyser (Agilent Technologies, Santa Clara, CA) with the Seahorse XFp Cell Mito Stress Test Kit (Agilent Technologies) in accordance with the manufacturer’s protocol. Briefly, cells were seeded into an Agilent Seahorse XFp well plate at a density of 1.5 × 10^5^ cells/mL and kept in a culture medium in the incubator for 24 h. While cells were near confluent, the culture medium was replaced with experimental medium for 36 h. One hour before the measurement, the medium was washed again and replaced with Seahorse XF Assay Medium supplemented with 2 mM l-glutamine, 1 mM sodium pyruvate, and 10 mM glucose, and cells were placed in a non-CO_2_ incubator. The kit components that had to be calibrated for final concentrations in the wells were 2 μM oligomycin, 1 μM carbonyl cyanide- 4-phenylhydrazone (FCCP), 0.5 μM antimycin A, and 0.5 μM rotenone by using a loaded assay cartridge. After baseline measurement, preloaded inhibitors were released consecutively into each well in a calibration chamber. The machine recorded oxygen concentration in pmol/min in every 4 min. The oxygen consumption rate (OCR) and extracellular acidification rate (ECAR) were normalized to total cellular protein content. The total protein concentration was determined by a standard colorimetric protein assay after each experiment.

### CPP preparation

CPPs were prepared by mixing CaCl_2_ (final concentration was 5 mM), NaH_2_PO_4_ and Na_2_HPO_4_ in 1:2 proportion (final concentration was 6 mM) in 25 mL of regular DMEM supplemented with 5% fetal bovine serum and 1% streptomycin/penicillin. These concentrations were adopted from a previous study^26^. This medium was incubated at 37 °C for 24 h and centrifuged at 16,000×*g* for 2 h. Subsequently, the supernatant was removed and precipitated CPPs were mixed with 5 mL of regular DMEM containing 10% FBS to make a CPP suspension that was later used as treatment.

### CPP-induced cell viability and cytotoxicity

DolKT-1 cells were incubated with or without CPPs, and CPPs in combination with 2 mM magnesium for 24 h. The WST-1 assay was performed to measure cell viability as stated above. To avoid the background of CPP turbidity, we also measured absorbance at a wavelength of 630 nm and subtracted it from the absorbance at a wavelength of 480 nm. A cytotoxicity assay was performed for CPP-exposed cells for 24 h by following the same protocols described above.

### Statistical analysis

All statistical analyses were performed with GraphPad Prism (ver., 5.0, https://www.graphpad.com). Data are presented as the mean ± standard error of the mean. One-way analysis of variance (ANOVA) followed by the Newman–Keuls multiple-comparison test was performed for all one-factor data to compare values in the control medium with those intervention groups. Comparison of two groups was performed using Students *t* test (parametric).

## Supplementary Information

Supplementary Information.

Supplementary Video 1.

## Data Availability

The data that support the findings of this study are available from Kagawa University but restrictions apply to the availability of these data, which were used under license for the current study, and so are not publicly available. Data are however available from the authors upon reasonable request and with permission of Kagawa University.
