# Supplementary material for: Possible contribution of phosphate to the pathogenesis of chronic kidney disease in dolphins
Source: Sci Rep. 2023 Mar 29;13:5161. doi: 10.1038/s41598-023-32399-6 (PMC10060237; doi:10.1038/s41598-023-32399-6)
Supplement: Supplementary file 1 — Supplementary Information. [file 41598_2023_32399_MOESM1_ESM.pdf]

Possible contribution of phosphate to the pathogenesis of chronic kidney disease in dolphins

Nourin Jahan[1], Hiroyuki Ohsaki[2]*, Kiyoko Kaneko[3]*, Asadur Rahman[1], Takeshi Nishiyama[4], Makoto Koizumi[5], Shuichiro Yamanaka[6], Kento Kitada[1], Yuki Sugiura[7], Kenji Matsui[6], Takashi Yokoo[6], Takayuki Hamano[8,9], Makoto Kuro-o[10], Takuya Itou[11], Miwa Suzuki[12], Keiichi Ueda[13], Akira Nishiyama[1]**

[1]Department of Pharmacology, Faculty of Medicine, Kagawa University, Kagawa, Japan

[2]Department of Medical Biophysics, Kobe University Graduate School of Health Science, Kobe, Japan

[3]Faculty of Pharmaceutical Sciences, Teikyo Heisei University, Tokyo, Japan

[4]Prime Hospital Tamashima, Kurashiki, Japan

[5]Laboratory Animal Facility, Research Center for Medical Sciences, Jikei University School of Medicine, Tokyo, Japan.

[6]Division of Nephrology and Hypertension, Department of Internal Medicine, The Jikei University School of Medicine, Tokyo, Japan.

[7]Multiomics Platform, Center for Cancer Immunotherapy and Immunobiology, Kyoto University Graduate School of Medicine, Kyoto, Japan.

[8]Department of Nephrology, Nagoya City University Graduate School of Medical Sciences, Nagoya, Japan.

[9]Department of Nephrology, Osaka University Graduate School of Medicine, Suita, Japan

[10]Division of Anti-Aging Medicine, Center for Molecular Medicine, Jichi Medical University, Tochigi, Japan.

[11]Nihon University Veterinary Research Center, Nihon University, Fujisawa, Japan

[12]Department of Marine Science and Resources, College of Bioresource Sciences, Nihon University, Fujisawa, Japan.

[13]Okinawa Churashima Foundation, Kunigami-gun, Japan.

*Hiroyuki Ohsaki and Kiyoko Kaneko contributed equally to this work.

**Corresponding Author:

Akira Nishiyama, MD, PhD

Department of Pharmacology, Faculty of Medicine, Kagawa University

1750-1 Ikenobe, Miki-cho, Kita-gun, Kagawa 761-0793, Japan

Phone: +81 87 891 2125

Fax: +81 87 891 2126

E-mail: nishiyama.akira@kagawa-u.ac.jp

Supplementary Figure S1

A

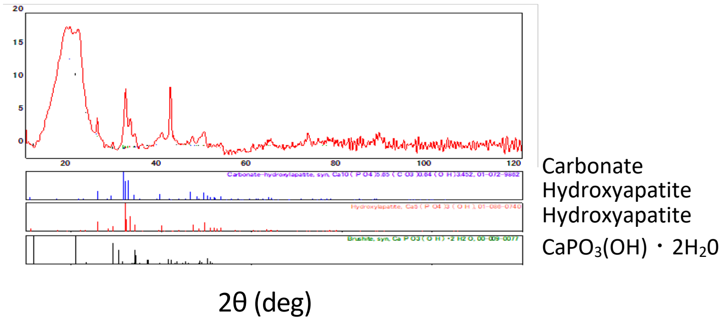

B

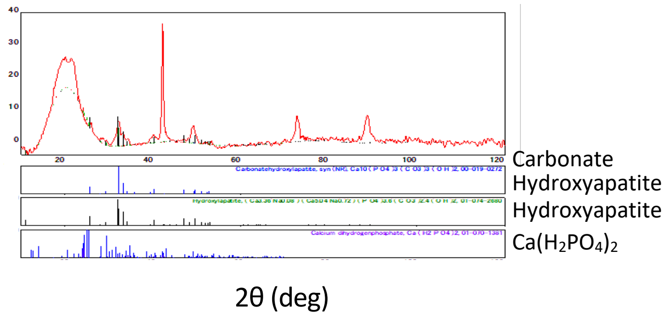

C

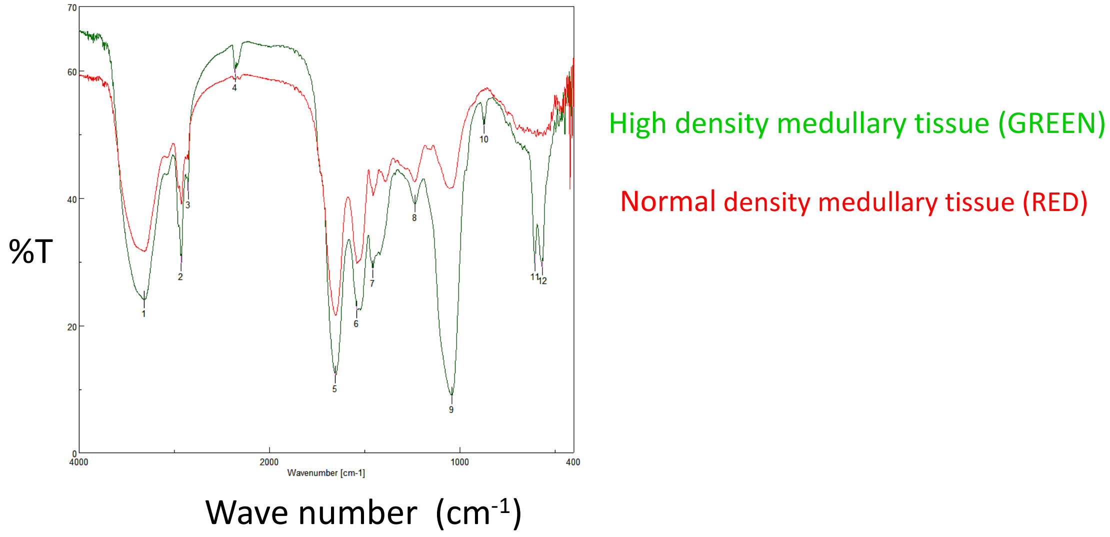

Supplementary Figure S2

A

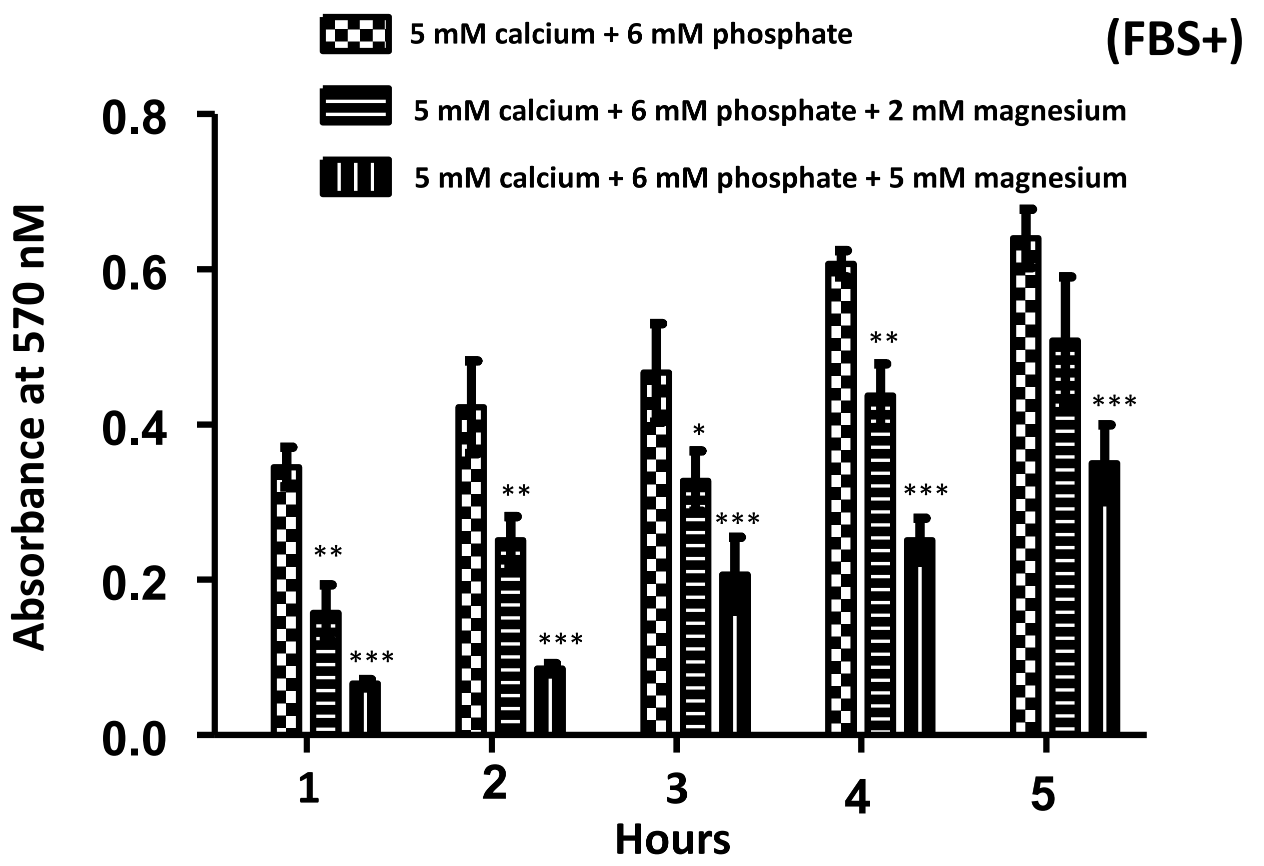

B

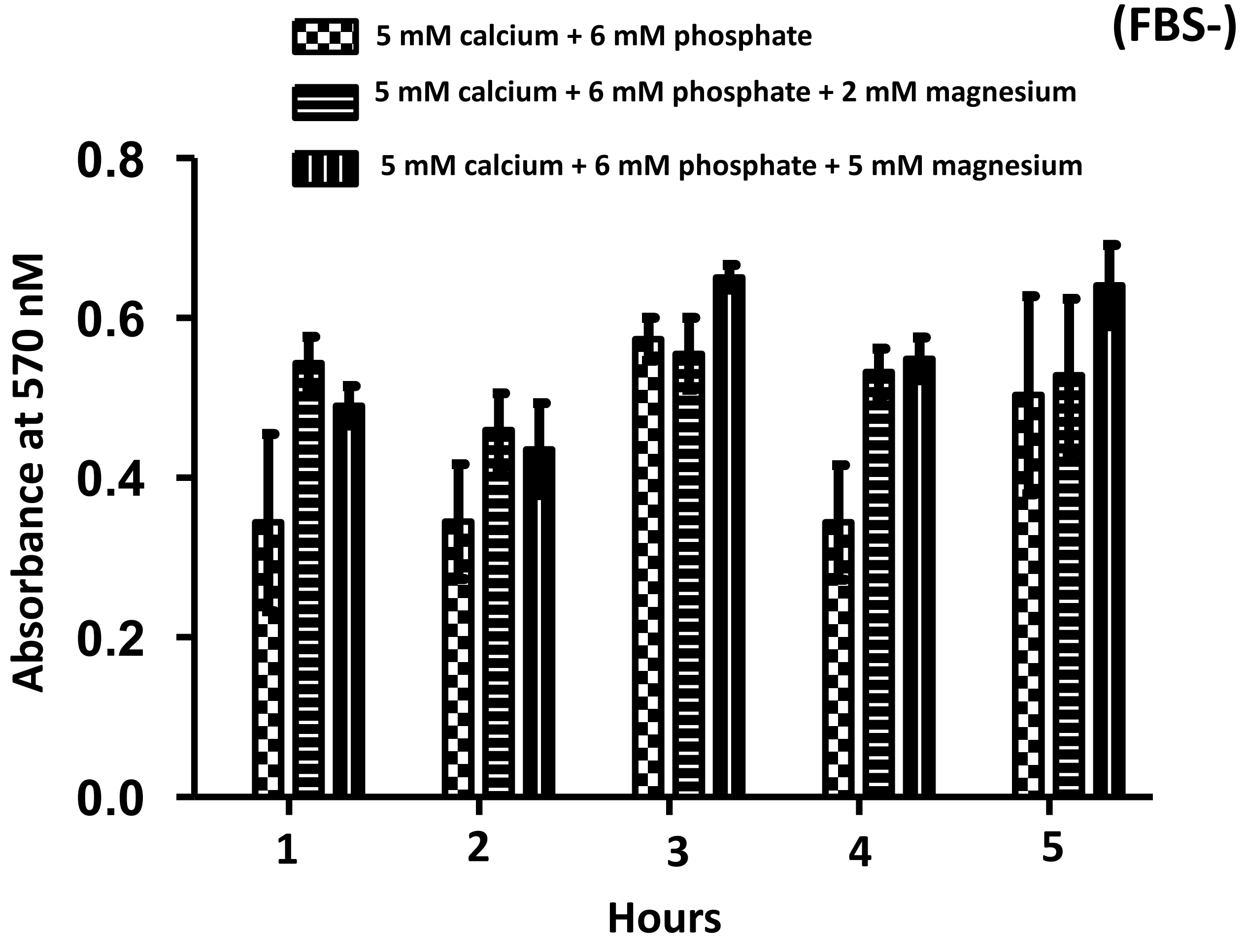

Supplementary Figure S3

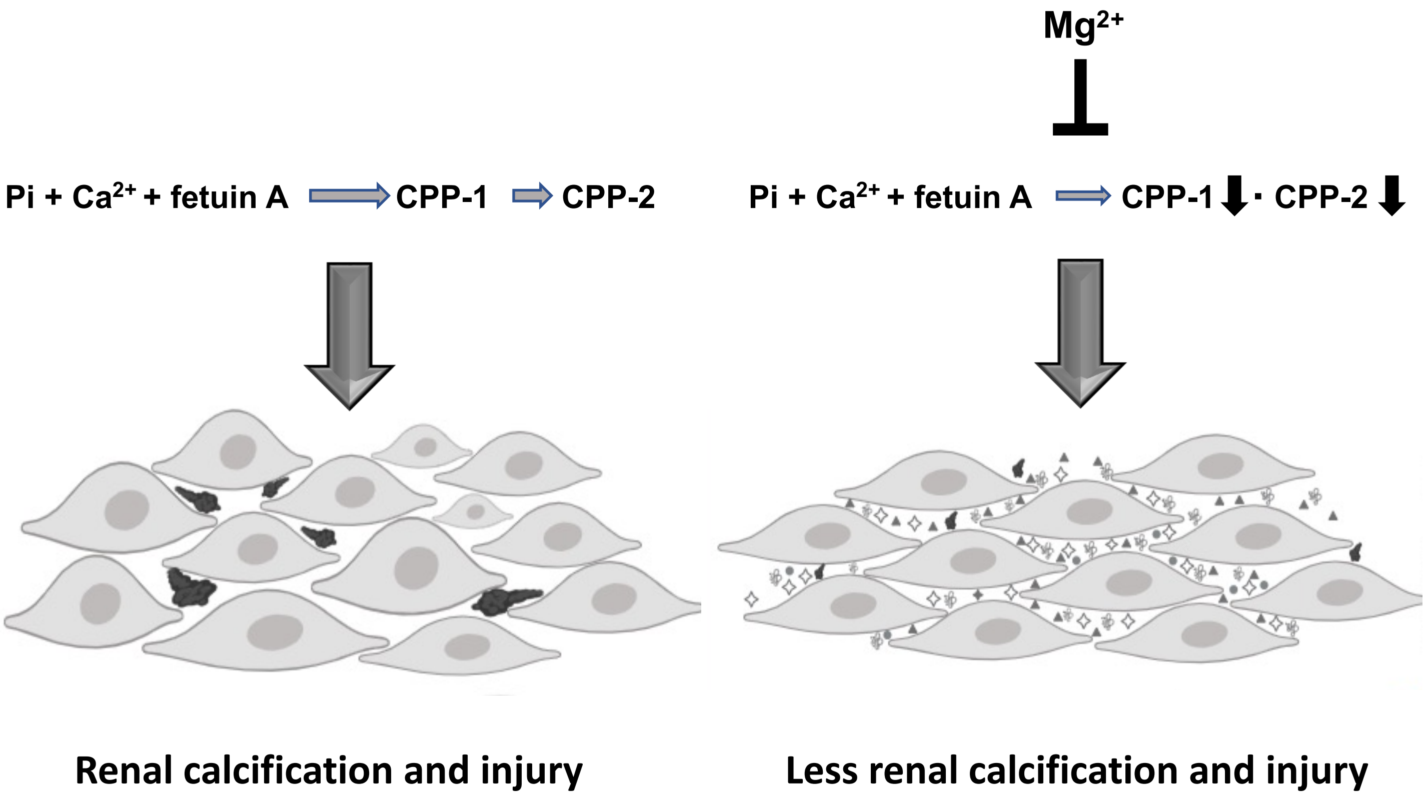

Supplementary Figure Legends

Supplementary Figure 1

Micro area X-ray and R spectroscopy.

Representative X-ray diffraction pattern of reniculi in medullary tissue of a high-density area (A) and a normal density area (B). Several points were analyzed using microbeam X-rays. The X-ray diffraction pattern was recorded with an X-ray goniometer and is represented in intensity as a function of twice the diffraction angle (2θ) curve. The vertical line shows the intensity of X-ray diffraction. Four retrieved standard substances in the JCPDS database are shown at the bottom. Microarea X-ray analysis showed that high-density area was composed of hydroxyapatite (calcium phosphate). A representative infrared spectroscopy (IR) pattern (C). After X-ray diffraction, tissues were analyzed with IR spectroscopy. Absorption of reniculi tissue of the high-density area was observed at 1457, 1040, 873, 606, and 567 cm^{-1} (green line). When the absorption wave data were compared with reported IR data, the renal tissue was shown to be composed of hydroxyapatite. However, in tissues of the normal density area, absorption at 1040 cm^{-1} was smaller, and those at 873, 606, and 567 cm^{-1} was not observed (red line).

Supplementary Figure 2

CPPs formation in DoIKT-1 cells.

The absorbance of high phosphate (5 mM) and high calcium (6 mM) medium supplemented with various magnesium concentrations (2 or 5 mM) in the presence of FBS was measured at 570 nm (A). OD values were normalized with the reference medium at each time point. n=6 for each group. Similarly, the absorbance of the high phosphate (5 mM) and high calcium (6 mM) medium supplemented with various magnesium concentrations (2 or 5 mM) in the absence of FBS was measured at 570 nm

(B). OD values were normalized with the reference medium at each time point. n=6 for each group. *P<0.005, **P<0.01 and ***P<0.001 vs. 5 mM phosphate + 6 mM calcium. Dolphin proximal tubular, DolKT-1. FBS, fetal bovine serum. OD, optical density.

Supplementary Figure 3.

Working hypothesis

Increased phosphate may bind with calcium and serum protein, fetuin-A to form CPPs. CPPs cause cellular injury which may result in renal calcification. Magnesium may decrease the formation of CPPs and attenuate the progression of chronic kidney disease in dolphins. CPP, calciprotein particles.

Supplementary Movie Data

Movie of the reniculi taken using micro-CT

File name: Micro-CT

Supplementary Table S1. Serum parameters.

Date	creatinine (mg/dL)	BUN (mg/dL)	calcium (mg/dL)	phosphorus (mg/dL)	potassium (mEq/L)	sodium (mEq/L)
November 28, 2002	1.4	73			3.9	155
April 17, 2004	1.6	52	9.2	4.4		
May 28, 2006	1.6	47			3.1	152
May 10, 2008	1.8	49			3.3	153
April 08, 2011	1.5	51	8.6	4.9	3.7	158
April 17, 2013	1.5	49	8.2	5.0	4.1	157
June 17, 2015	1.3	68				
Sep 03, 2017	1.1	103	8.8	6.5	3.2	162
February 13, 2018	0.7	120	9.4	7.2	3.9	164
March 07, 2018	0.8	121	8.8	8.5	3.7	164
March 19, 2018	0.7	153	8.1	5.7	3.4	151

BUN, blood urea nitrogen
