# Supplementary figures and images for: Possible contribution of phosphate to the pathogenesis of chronic kidney disease in dolphins
Source: Sci Rep. 2023 Mar 29;13:5161. doi: 10.1038/s41598-023-32399-6 (PMC10060237; doi:10.1038/s41598-023-32399-6)

## Slide 1
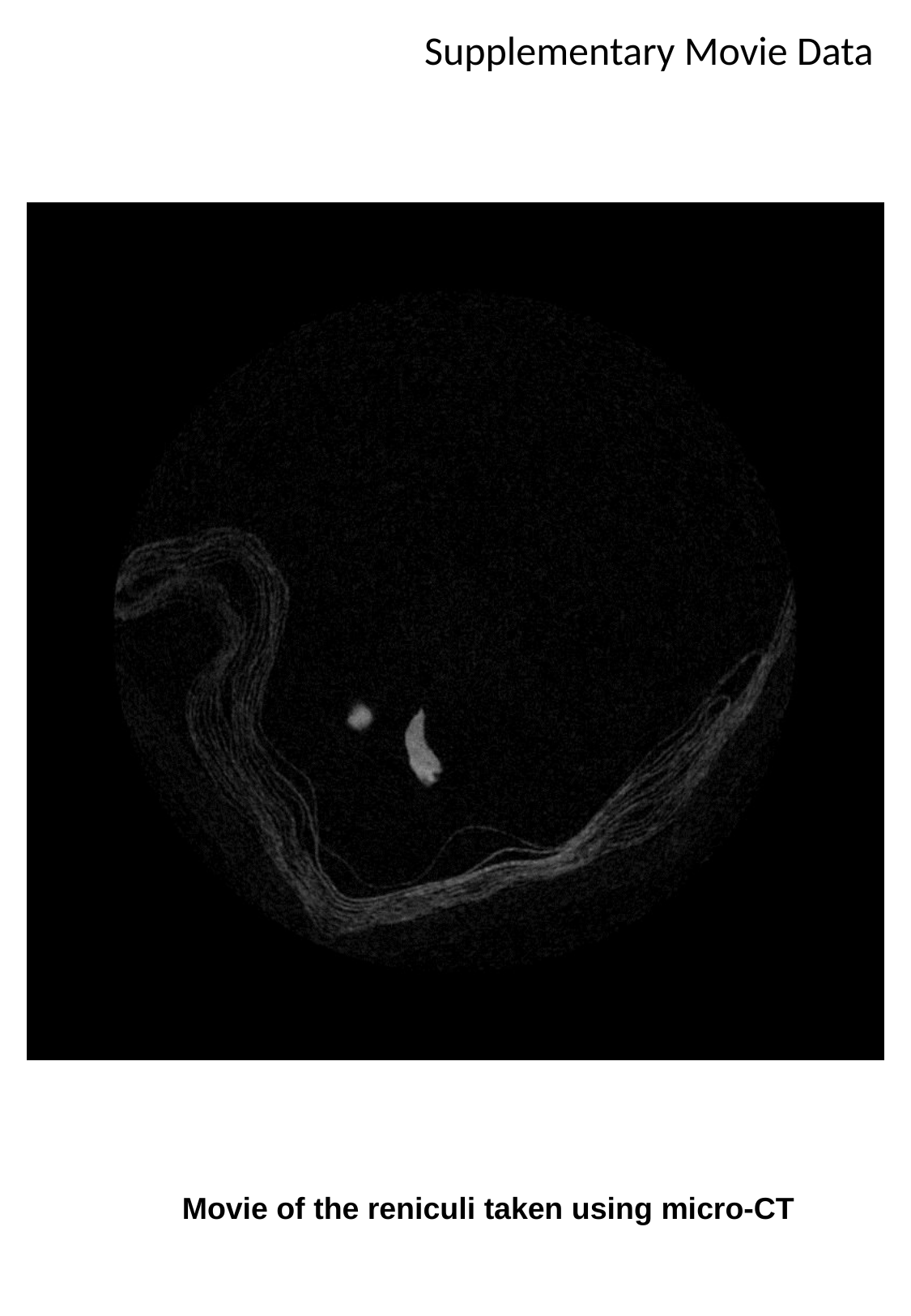

Supplementary Movie Data
Movie of the reniculi taken using micro-CT

Supplement: Supplementary file 2 — Supplementary Video 1. [file 41598_2023_32399_MOESM2_ESM.pptx]
